# Perennial transmission of malaria in the low altitude areas of Baringo County, Kenya

**DOI:** 10.1186/s12936-017-1904-y

**Published:** 2017-06-17

**Authors:** Collince J. Omondi, Daniel Onguru, Lucy Kamau, Mark Nanyingi, George Ong’amo, Benson Estambale

**Affiliations:** 1 Department of Zoological Sciences, Kenyatta University, P.O. Box 43844, Nairobi, 00100 Kenya; 2 School of Health Sciences, Jaramogi Oginga Odinga University of Science and Technology, P.O. Box 210, Bondo, 40601 Kenya; 3 Department of Public Health, Pharmacology and Toxicology, University of Nairobi, P.O. Box 30197, Nairobi, 00100 Kenya; 4 School of Biological Sciences, University of Nairobi, P.O. Box 30197, Nairobi, 00100 Kenya

**Keywords:** Malaria, Children, RDT, Riverine

## Abstract

**Background:**

Malaria causes the greatest public health burden in sub-Saharan Africa where high mortality occurs mainly in children under 5 years of age. Traditionally, malaria has been reported mainly in the lowlands endemic regions of western Kenya, while the highlands of the Rift Valley have been relatively free except for the sporadic epidemics in some areas. Baringo County is located in the Kenyan highlands. The county generally experiences seasonal transmission of malaria. A few hotspots which experience continuous malaria transmission in the county do however exist. The objective of this study was to assess malaria infection status and identify areas with continuous transmissions with a view to mapping out probable transmission hot spots useful in mounting focused interventions within the county.

**Methods:**

Systematic sampling was employed to identify 1668 primary school pupils from fifteen primary schools located in 4 ecological zones (lowland, midland, highland and riverine) of three sub-counties of Baringo. Finger prick blood sampling was done every 4 months (during the dry season in February/March, after the long rains in June/July and short rains in November 2015). Malaria occurrence was tested using rapid diagnostic test kit (CareStart HRP-2 Pf). Microscopic examination was done on all RDT positive and 10% of negative cases.

**Results:**

A total of 268 (16.1%), out of 1668 pupils tested positive for *Plasmodium falciparum* by RDT; 78% had a single episode, 16.8% had 2 episodes, 4.9% had 3 episodes and 0.4% had 4 episodes. The riverine zone had the highest malaria cases (23.2%) followed by lowlands (0.9%). No malaria cases were detected in the midland zone while highland zone recorded only few cases during the third follow up. Up to 10.7% of malaria cases were reported in the dry season, 2.9% during the long rains and 5.7% in short rains season.

**Conclusions:**

Malaria infection was prevalent in Baringo County and was mainly restricted to the riverine zone where transmission is continuous throughout the year. High malaria prevalence occurred in the dry season compared to the wet season. Even though malaria transmission is relatively low compared to endemic regions of Kenya, there is a need for continued monitoring of transmission dynamics under changing climatic conditions as well as establishing expanded malaria control strategies especially within the riverine zone which would include an integrated mosquito control and chemotherapy for infected individuals.

## Background

In spite of substantial efforts towards malaria control in many endemic countries, the disease continues to be an important vector-borne parasitic disease worldwide [1]. Globally, 3.2 billion people are estimated to be at risk of malaria while 214 million cases resulted in 438, 000 deaths in the year 2015 [2]. About 88% of these deaths occurred in sub-Saharan Africa where young children are the most affected [2, 3]. In Kenya, malaria is the leading cause of death in children under 5 years of age [4, 5]. Transmission pattern in the country is quite diverse with endemic regions (western part of the country and the coastal region) experiencing continuous transmission throughout the year. The western highlands of Kenya experience both seasonal and epidemic malaria during long rains, while the arid and semi-arid areas including Baringo County experience seasonal malaria transmission which intensifies during and just after the rains [4].

Decline in malaria burden in most regions of the world has been linked to intensification of high impact control interventions [6, 7]. Currently, early treatment with effective anti-malarial drugs which is the main life-saving intervention is threatened by intensification of the growing resistance of parasites to the existing therapies. Based on this, the need to employ new, effective and sustainable strategies towards malaria control and management is of utmost importance.

Understanding the current incidence and transmission patterns of malaria can positively influence the choice of control tools deployed in different epidemiological zones. Baringo County is considered a low malaria transmission area, with seasonal patterns [4, 7]. Such areas are mostly characterized by pockets of transmission, which are usually intense in nature [8]. Moreover, malaria transmission becomes more focal in nature as it declines [9]. These features necessitate extensive malaria screening to map out the possible existence of sustained foci of transmission, necessary for the estimation of the actual malaria burden [10]. Unfortunately, few studies are usually conducted in low transmission areas with little resource allocation, leading to scarcity of information, hence no interventions or inappropriate ones are put in place to curb the malaria transmission [11].

Information on infection risks and clinical epidemiology in Baringo County is limited [7] and only documented as exhibiting seasonal transmission which intensifies during the short rains and just after the long rains. This may not take into account the existence of hotspots that may be experiencing malaria transmission throughout the year. The objective of this study was therefore to determine malaria point prevalence within three sub-counties (Marigat, Baringo Central and Baringo North) of Baringo County, with a view to identifying probable transmission hot spots, useful in mounting focused interventions.

## Methods

### Description of the study area

**Fig. 1:**
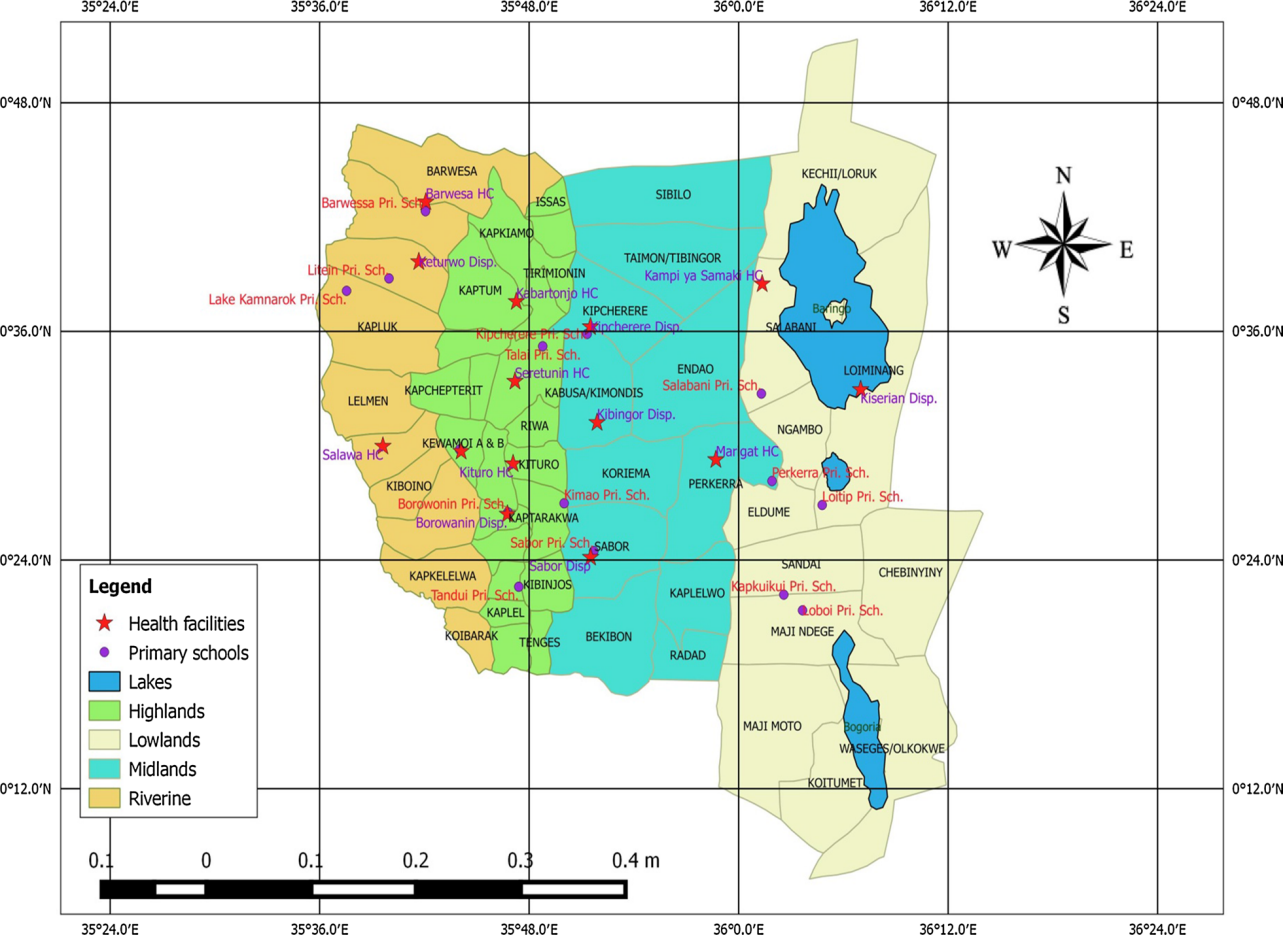
Map of study area showing the four ecological zones studied within three sub counties; only selected schools are shown (Baringo North, Central and South)

The study was conducted in three sub-counties (Marigat, Baringo North and Baringo Central) of Baringo County between February 2015 and February 2016. Baringo County is located within the Rift Valley and lies between longitude 35.602°–36.277°E, and latitude 0.541°–0.723°N at altitudes ranging between 870 and 2499 m above sea level (asl). The study area represents arid and semi-arid parts of Baringo County. The study area was divided into four strata running parallel to each other in a North–South direction. These strata were identified based on the hydrology, soil types, rainfall patterns, vegetation cover and altitude (Fig. 1). The four zones included; lowlands (900–1000 m asl), midlands (1000–1500 m asl), highlands (1500–2300 m asl) and riverine (1000–1200 m asl). The lowland zone lies to the east of the study area and receives mean annual rainfall of about 650 mm with temperatures ranging between 30 and 37 °C. The area is characterized by both lakes (Lake Baringo and Lake 94) and perennial rivers (Perkerra and Molo) with poor drainage soil types. The midland zone is characterized by well drained soil types and is interspersed with dry river beds that flow only after heavy rains. The highland zone receives an average rainfall that ranges between 1000 and 1500 mm per year with well drained soil types, while the riverine zone borders the Kerio Valley to the west of the study area. Lake Kamnarok is the major water body within this zone where the soil is poorly drained and prone to flooding.

According to 2009 Kenya national population census, Baringo County has a population of 555,561 people distributed in 11,075.3 km^2^. The temperatures range from a minimum of 10 °C to a maximum of 37 °C in different parts of the county. The region has two distinct weather patterns with the cold months (June and July) and the hot months (January and February). It experiences two rainy seasons, March to June (long rains) and November (short rains). It receives between 1000 and 1500 mm of rainfall annually in the highlands and 600 mm in the lowlands. Malaria transmission in this region is seasonal with peaks during the rainy season. The current incidence of malaria is about 12% based on outpatient visits to local health facilities [12]).

### Study design

The study was longitudinal in design where 15 public day primary schools were selected from the four zones based on their close proximity to mosquito breeding (aquatic) habitats. These breeding sites had been identified by a parallel entomological study by the same team, and were equitably spatially distributed across the study area. From these, the school closest to a breeding site included in the study was conveniently selected, and based on the populations of the respective schools, 15 were adequate for the desired sample. From these schools, at least 1540 pupils aged between 5 and 15 years were required for the study, was estimated using a formula proposed by Cochran [13]. The study, however, enrolled 1668 pupils.

$$ n = \frac{{Z^{2} pq}}{{e^{2} }} \Rightarrow n = \frac{{1.96^{2} \times 0.5 \times 0.5}}{0.05 \times 0.05} = 385 \Rightarrow 385 \times 4(zones) = 1540 $$

where: *n* is the sample size, *Z* is the value for the selected alpha level, e.g. 1.96 for (0.25 in each tail) a 95 percent confidence level. *p* is the estimated proportion of an attribute that is present in the population (0.5). *q* is 1 − *p*. (*p*)(*q*) are the estimate of variance. *e* is the acceptable margin of error for proportion being estimated (0.05).

Following parental or guardian informed consent, 1668 pupils were recruited to mitigate against possible loss during follow-ups (see Table 1). During baseline study (January/February 2015) and each of the follow-ups (June/July 2015, October/November 2015, and January/February 2016), CareStart™ HRP-2(Pf) (Cat No. G0140 RDT Access Bio) kits were used to test for the presence of parasite antigen in whole blood, according to the manufacturers’ instructions. Pupils were physically examined and vital signs (weight, body temperature general health) recorded. Pupils were also asked for history of medication for febrile illnesses in the previous 4 weeks, and only those without were enrolled. One finger prick blood sample (20 µl) was then collected for smears and RDT. Both thick and thin smears were prepared in the field on the same slide and stored for subsequent laboratory examination. In the laboratory, thin and thick blood films were stained using 10% Giemsa stain. They were then examined under (100×) oil immersion lens. Slide examination was carried out by two microscopists. For positive slides, number of parasites were counted against 200 leucocytes and quantified as parasite density/µl of blood. Slides were considered negative when no parasite was detected only after examining 100 microscopic fields… All individuals diagnosed positive for *Plasmodium* spp. were treated for malaria using the recommended drug (Artemether + Lumefantrine) by the nurse; the pupils were given the first dose instantly, and instructed to complete the remaining doses at home. The average monthly rainfall data was downloaded from IRI/LDEO climate data library from January 2015 to January 2016 [14].

**Table 1:** Summary of primary schools enrolled for the study; location and pupils tested

Ecological zones	Name of primary school	School code	School population	No. consented and tested
Riverine	Lake Kamnarok	P1	300	207
Riverine	Litein	P2	380	268
Riverine	Barwessa	P3	500	288
Highlands	Talai	P4	312	100
Highlands	Tandui	P5	491	95
Highlands	Kaptimbor	P6	694	58
Highlands	Borowanin	P7	224	31
Midlands	Kimao	P8	94	50
Midlands	Sabor	P9	228	83
Lowlands	Kapkuikui	P10	237	70
Lowlands	Loboi	P11	350	97
Midlands	Kipcherere	P12	360	46
Lowlands	Salabani	P13	215	119
Lowlands	Perkerra	P14	277	40
Lowlands	Loitip	P15	370	116
Total		15	5032	1668

Incidence was estimated as the number of cases per 1000 person-months at risk. When detecting cases, any pupil who tested positive for malaria or missed on the testing was considered out of the study and was not followed. Time at risk for each pupil was then calculated based on how long the pupil was followed during the entire study period. Those who tested positive at baseline survey were not included when calculating time at risk since they all had time zero at risk. The incidence rate was calculated at an interval of 4 months for a period of 8 months. Those who tested positive during the first follow up had 2 months time at risk each, while in the second follow up, each had 6 months at risk. Those who were followed throughout the study period each had 8 months time at risk. Date of testing during baseline and subsequent follow up was recorded to determine time interval between each surveys.

Data obtained was entered in MS Excel spreadsheet, cleaned and malaria incidence was analysed using STATA version 12 (Stata Corporation, College station, Texas, USA). R version 3.0.3 was used to calculate point prevalence at 95% confidence interval of proportions while frequencies and cross tabulation to determine asymptomatic cases were calculated using SPSS version 22.

### Ethical approval

Ethical approval to conduct the study was obtained from Kenyatta National Hospital and University of Nairobi Ethics and Research Committee (P70/02/2013). Authority to conduct research was granted by the Director of Health Services, Baringo County. Written informed consent was obtained from each parent or guardian, and only willing children were enrolled into the study.

## Results

### Malaria point prevalence

A total of 1668 primary school pupils were recruited and tested for malaria parasites during the baseline survey carried out in the dry season (January/February 2015) (Table 2). Of the total number tested, 175 (10.5%) (95% CI 9.1–12.1) pupils were positive for *P. falciparum* infections. The highest point prevalence was recorded from the riverine zone (22.8%) (95% CI 19.9–26.0) followed by the Lowlands (0.2%) (95% CI 0.01–1.5). Both midland and highland zones had no cases of *Plasmodium* species infections (Fisher’s exact test = 0.005).

**Table 2:** Malaria positive cases per zone

Zones	Baseline study		First follow up		Second follow up		Third follow up	
	Number tested	+ve n (%)	Number tested	+ve n (%)	Number tested	+ve n (%)	Number tested	+ve n (%)
Riverine	763	174 (22.8)	560	35 (6.5)	594	68 (11.4)	582	74 (12.7)
Lowland	442	1 (0.2)	396	0	319	2 (0.6)	321	1 (0.3)
Highland	284	0	256	0	224	0	257	5 (1.9)
Midland	179	0	160	0	141	0	131	0
Total	1668	175 (10.5)	1372	35 (2.6)	1278	70 (5.5)	1291	80 (6.2)

Fisher’s exact test = 0.005

During the first follow-up conducted towards the end of long rains (June/July 2015) the overall prevalence dropped from 10.5% (95% CI 9.1–12.1) to 2.6% (95% CI 1.8–3.6), although this difference was not statistically significant (Fisher’s exact test = 1.000). The number of pupils who were tested also dropped from 1668 to 1372 (17.7% loss to follow up). All positive cases were from the riverine zone giving a prevalence of 6.5%.

The second follow up was carried out during the short rains (October/November 2015). Malaria prevalence rose up to 5.5% (95% CI 4.3–6.9) from 2.6% (95% CI 1.8–3.6) (Fisher’s exact test = 0.551). The loss to follow up rose to 23.4%. The prevalence of malaria in the riverine zone was 11.4% (95% CI 9.1–14.4), while that in the lowland Zone was 0.6% (95% CI 0.1–2.5). No positive cases were reported in the highland and midland zones. The third follow-up was conducted immediately after the end of the El Niño rains (January/February 2016). The riverine zone recorded the highest prevalence of malaria infection (12.7%) (95% CI 5.0–7.7) compared to highlands (1.9%) (95% CI 0.7–4.7) and the Lowlands with 0.3% (95% CI 0.02–2.0). The overall point prevalence of malaria infection was 6.2% (95% CI 5.0–7.7). The midland Zone had no malaria cases during the entire period of the study.

In general, prevalence varied with altitude where the riverine zone recorded higher prevalence followed by the lowland and the highland (Fisher’s exact test = 0.005) (Table 1). *Plasmodium* species infection was slightly high during dry season compared to long rain season and short rain season.

### *Plasmodium* species infection episodes per child

During the baseline survey and the three subsequent follow-ups, a total of 268 (16.1%) pupils were positive for *P. falciparum* infection. Out of these, 209 (78.0%) pupils had a single episode of infection, 45 (16.8%) had 2 episodes, 13 (4.9%) had 3 episodes while 1 (0.4%) had 4 episodes of malaria. No death due to malaria was reported during the surveillance period.

### Malaria incidence

The overall malaria incidence within the entire study area was estimated at 6/1000 person-months. This indicated that at least six people out of 1000 were at risk of *P. falciparum* infection every month. Malaria incidence varied greatly when categorized into geographical zones, with the riverine recording the highest (RR = 40.2 (95% CI 7–1623). While the highland and midland zones recorded zero incidence during the entire study period, the lowland had an incidence of 0.5/1000 person-month. The riverine zone had ant incidence of 14/1000 person-months (Table 3).

**Table 3:** Incidence rate by ecological zone

Zone	Person-month	New cases	Incidence rate/1000	95% Conf. interval		RR
				Lower limit	Upper limit	
Highland	1928	0	0	–	–	
Lowland	2084	1	0.5	0.0000676	0.0034065	
Midland	1060	0	0	–		40.2
Riverine	3310	47	14.2	0.0106686	0.0188986	
Total	8382	48	57	0.0043155	0.007599	

Rate ratio 40.2 (95% CI 7–1623)

The risk of *Plasmodium* species infection was further analysed by age groups; 5–9 and 10–15 years old. Results indicated that those in the 10–15 years category were at a greater risk of *P. falciparum* infection (7/1000 person-months) when compared to 5–9 years age group (4/1000 person-months) although this was not statistically significant (RR = 1.63 (95% CI 0.9–3.1). Similarly, the rate of *Plasmodium* species infection between males and females was not significantly different within the study area. IRR = 1.02 (95% CI 0.55–1.88) (Table 4).

**Table 4:** Incidence rate by age group and gender

	PM	Cases	Incidence rate/1000	95% CI		RR
				Lower limit	Upper limit	
Age (years)						
5–9	4332	19	4.386	2.798	6.876	–
10–15	4056	29	7.150	4.969	10.29	1.63
Total	8388	48	5.722	4.312	7.594	
Gender						
Males	4236	24	5.666	3.798	8.453	–
Females	4152	24	5.780	3.874	8.624	1.02
Total	8388	48	5.722	4.312	7.594	

*PM* person-month, *RR* rate ratio

### Prevalence of asymptomatic *Plasmodium* species infection

Asymptomatic *Plasmodium* species infection (infected individuals with no clinical signs or symptoms) was determined using both RDT and microscopy. During baseline survey, 61.1% (95% CI 0.53–0.68) of the positive cases by RDT were asymptomatic. The proportion of asymptomatic cases were relatively high in both first follow up and second follow up, 65.7% (95% CI 0.48–0.8) and 62.9% (95% CI 0.5–0.7), respectively. During the third follow up, 48.9% of the positive cases were asymptomatic. Pearson Chi square was used to determine the possible association between asymptomatic *Plasmodium* species infection to gender and age groups. Males were slightly more asymptomatic than females, however, the results indicated no statistically significant difference (Pearson χ^2^ = 2.8885, df = 3, p = 0.409). Similarly, there was no difference of asymptomatic *Plasmodium* species infection between children aged 5–9 and 10–15 years (Pearson χ^2^ = 0.6746, df = 3, p = 0.879) (Table 5).

**Table 5:** Asymptomatic cases by gender

	Survey				$\chi_{3}^{2}$	Pr
	Baseline	FUP	SUP	TUP		
Gender						
Female	44	12	13	18	2.8885	0.409
Male	60	11	27	20		
Age(years)						
5–9	50	11	19	21	0.6746	0.879
10–15	54	12	21	17		

*FUP* first follow up, *SUP* second follows up, *TUP* third follows up

### Rainfall pattern in relation to malaria transmission

**Fig. 2:**
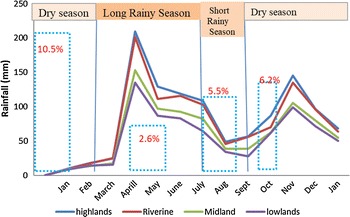
Malaria transmission in relation to rainfall pattern for the year 2015 (http://iridl.ldeo.columbia.edu/SOURCES/.UCSB/.CHIRPS/.v2p0/.monthly/#expert). *OMP* overall malaria prevalence, *DS* dry season, *LR* long rain, *SR* short rain

Baringo County has a bimodal rainfall pattern with the long rains falling between April and July, and the short rains between August and November. The dry season occurs between December and March. Malaria transmission was highest during the months of January/February followed by October/November and lowest during June and July (Fig. 2).

## Discussion

The findings of the present study, revealed the presence of *P. falciparum* infections in parts of the Rift Valley highlands not previously reported, particularly in the lower altitude areas, although highland malaria has widely been reported [15–18]. This study found that *Plasmodium* species infections are mainly restricted to the riverine zone, with *P. falciparum* as the main species. While it is not possible for this study to describe the reasons for the observed distribution, it is likely that ecological and environmental factors, including altitude, vegetation, terrain, water bodies, rainfall, temperature, humidity and vector abundance could have played a role, as previously reported [19–23]. Human activity, especially land use, has also been reported to influence malaria transmission patterns [20], which is a key consideration for the interpretation of these results given the diversity in land use across the four zones in the study area.

*Plasmodium* species infection within lowland was lower than that of the riverine zone, although both zones bear nearly similar environmental characteristics, which are different from the midland and highland zones. A likely explanation for this difference is the different control strategies used within these two zones. The lowland zone has had more interventions, especially the use of treated bed nets and indoor residual spray of houses to control leishmaniasis [24]. In addition, there is a low access to health services in the riverine zone due to the few, distant and ill-equipped health facilities, which are not connected to proper road networks.

The present study findings indicate higher *Plasmodium* species infection during the dry season compared to the wet season. This is similar to other study findings, which linked high transmission during dry season to peri-domestic crop production and household levels [25, 26]. The lowest prevalence was recorded towards the end of long rains while during short rainy season, the prevalence slightly increased. Low malaria cases during long rains is due to flushing off of malaria vector breeding sites which may further lead to a decrease in larval population [27, 28].

Although the overall malaria prevalence within Baringo County is low (2.6–10.5%) compared to endemic regions of Kenya (over 20%) [29], the riverine zone exhibited higher rates of malaria transmission throughout the study period. Transmission in both dry and wet seasons might also be indicative of a perennial pattern, which contrasts previous findings that described malaria transmission within Baringo County as seasonal [4, 7]. This might point the possible emergence of a hot spot within the county.

The entire study area indicated low risk of *Plasmodium* sp. infection, but the risk greatly varied with ecological zones. The riverine zone recorded the highest risk while lowlands, highlands and midlands recorded least or no risk of infection. These findings are consistent with other studies which demonstrated micro-epidemiological variations in malaria exposure especially in low transmission areas [7, 9, 30]. The results further indicated that children aged 10–15 years were slightly more at risk than those aged 5–9 years old, although not statistically different, but points to agreement with other studies which have reported more susceptibility among the age group of 10–15 years than those aged 5–9 years [31, 32]. Infection rate for males and females was similar, in contrast to other studies which indicated that females were more at risk for malaria infection [32–34]. It is however important to note that using RDT to diagnose malaria is likely to miss low density infection and therefore the data presented mainly portrays the incidence of detectable infections, and not necessarily the incidence of new infections.

The proportion of asymptomatic *Plasmodium* species infections by RDT was high during the entire study period except in the third follow up. Microscopy also indicated that over 50% of the confirmed cases during the study had no clinical symptoms, supporting previous findings showing that school-age children represent the group with high cases of asymptomatic malaria [35, 36]. Asymptomatic cases are usually common in high transmission areas where continuous exposure leads to development of partial immunity in children [37]. Baringo County malaria transmission has been considered to be seasonal and parasite prevalence is usually below 5% [4, 38]. However, the presence of asymptomatic individuals particularly within riverine zone may point a continuous malaria transmission rather than seasonal.

## Conclusions

This study reports the occurrence of malaria in Baringo County, which is confined mainly to the lower altitude riverine areas, and which exhibit perennial transmission. High malaria prevalence is recorded during the dry season in the months of January to March. There is therefore a need for sustained and expanded malaria control strategies especially within the riverine zone of the county.
